# A unidimensional short form of the Beck Hopelessness Scale (BHS-7) derived using item response theory

**DOI:** 10.1038/s41598-024-56792-x

**Published:** 2024-03-12

**Authors:** Tyrone B. Pretorius, Anita Padmanabhanunni

**Affiliations:** Department of Psychology, University of the Western Cape, Cape Town, South Africa

**Keywords:** Psychology, Quality of life

## Abstract

The Beck Hopelessness Scale (BHS) is the most widely used measure of hopelessness, a key psychological construct linked with various mental health outcomes. In clinical settings, the BHS has proven a reliable tool for assessing hopelessness; however, there has been debate regarding the tool’s internal consistency among non-clinical populations. Most studies assessing the dimensionality of the BHS have relied on the use of classical test theory (CTT). The length of the BHS has also prompted concerns over its practicality. The BHS-9 was developed to address these critiques and formulated based on psychiatrically hospitalized adult patients. The current study investigates the dimensionality of the BHS-9 among a non-clinical sample using item response theory (Mokken scale analysis and Rasch) and CTT. The results confirm that the BHS-9 is essentially unidimensional. However, a salient finding was that Item 6 violated invariant item ordering. An exploratory factor analysis of the remaining eight items found that the items accounted for 48.05% of the variance. Further exploratory factor analyses, removing one item at a time, showed that the removal of item 18 would increase variance explained > 50%. The revised BHS-7 was found to be unidimensional and maintained strong internal consistency and criterion-related validity. This revised tool effectively captures the essence of hopelessness among a non-clinical population and presents a more refined option for the assessment of this construct.

## Introduction

Hopelessness is a significant psychological concept characterized by negative perceptions about oneself and one's future^1^. This state of mind is marked by pessimistic expectations and an accompanying negative emotional state in which solutions to personal problems seem elusive or entirely out of reach. Hopelessness is a key feature in models of depression^2,3^ and has been theorized to be the bridge between cognitive vulnerability and depressive disorders. Beck^1^ conceptualized hopelessness as a cognitive attributional style that leads depressed individuals to view suicide as a viable option to deal with problems that are subjectively appraised as unsolvable. The construct of hopelessness has been framed within the cognitive-vulnerability stress model^2,4^, which posits that some individuals display vulnerability to negative affective states owing to a tendency to attribute negative life events to internal (rather than external) factors, view these as stable (rather than temporary) conditions, and perceive them as global (instead of specific) occurrences. A significant empirical evidence base has emerged in support of Beck’s theory, confirming that hopelessness is one of the most salient risk factors for suicidal ideation and behavior among clinical and non-clinical populations^5–7^.

A 10-year longitudinal study of depression patients^5^ in the US found that hopelessness was a significant risk factor for suicidal ideation. Similarly, a 10-year cohort study^8^ of first admission patients with psychosis in the US reported that hopelessness confers suicide risk above and beyond a history of attempted suicide among this population group. In a nationally represented study, Perczel Forintos and colleagues^9^ measured the level of hopelessness among the Hungarian population with the application and psychometric analysis of the short version of the Hopelessness Scale. These researchers^9^ reported that people who had attempted suicide in the year prior to the study had higher scores for hopelessness than those with a prior history of suicide attempts. A cross-sectional study assessing hopelessness and suicidal ideation among Iranian medical students^6^ found that after adjusting for demographic variables (e.g., age, gender, and marital status), hopelessness was independently associated with suicidal ideation. A meta-analysis of longitudinal studies^10^ investigating depression and hopelessness as predictors of suicidal ideation, attempts, and completion concluded that hopelessness was the strongest predictor of each of these factors.

Due to the well-established association between hopelessness and suicide, the assessment of hopelessness is recommended as a standard procedure in screening individuals who express suicidal thoughts^9,10^. In addition, assessing hopelessness is an integral component of thorough treatment plans for individuals with various mental health disorders^1^. Since its development in 1974, the Beck Hopelessness Scale (BHS)^1^ has emerged as the most widely used standardized measure of hopelessness. The scale has been translated into a range of languages, including German^11^, Yoruba^12^, Chinese^13^, Italian^14^, Japanese^15^, and isiXhosa^16^. The original version of the BHS comprises 20 items with a true-or-false response format. The scale consists of an affective, motivational and cognitive factor. Responses are summed to generate a severity rating from 0 to 20, with higher scores indicating a stronger presence of hopelessness.

Existing studies have reported differences in the factor structure of the scale in both clinical and non-clinical populations (see^17^ for a review). For example, Dyce^18^ investigated the factor structure of the scale among a sample of outpatients and found that Beck’s three factor model had the best fit. Szabó and colleagues^19^ assessed the construct validity of the BHS in a large clinical sample using confirmatory and exploratory factor analytic techniques and concluded that a bi-factor model was the best fit. In a longitudinal study of cancer patients, Spangenberg and colleagues^20^ reported that exploratory factor analysis (EFA) yielded a two-factor solution. Similarly, non-clinical studies have reported variations in the factor structure of the BHS. For example, Pompili and colleagues^14^, using confirmatory factor analysis (CFA), investigated the factor structure of the BHS among Italian students and reported that a two-factor solution best fit the data. A study of the factor structure of the BHS among the Colombian general population^21^ using CFA supported the original three-factor model. Hanna and colleagues^22^ used CFA to assess the psychometric properties of the BHS among students in the United Kingdom and reported that a one factor model was the best fit. Furthermore, there have been variations in the reliability of the scale in non-clinical samples (e.g., α = 0.65^23^; α = 0.81^21^; α 0.87^11^). The variations in internal consistency may stem from differences in the way hopelessness is expressed and experienced among non-clinical populations compared to populations with mental health disorders^21,24^. Some studies have found lower reliability coefficients among non-clinical samples^23^ than among clinical samples^19^. As a consequence of these inconsistencies, there has been a call^22,24^ for more research into the factor structure of the BHS, particularly in non-clinical populations.

In addition to concerns about the factor structure and internal consistency of the BHS, the length of the scale has been regarded as unwieldy for practitioners and respondents^25^, leading to concerns over the tool’s practicality in certain assessment situations. Lengthy questionnaires may lead to respondent fatigue and reduce the quality of the data^25^. These concerns have prompted the development of shortened versions of the scale, comprising three^26^, four^27^, and nine items^25^, respectively.

These condensed versions of the BHS aim to retain the essential features of the original scale while offering a more efficient and practical alternative to assess hopelessness in various contexts. For example, Aish and Wasserman^27^ used CFA to report that a 4-item version of the BHS (comprised of Items 6, 7, 9, and 15) exhibited an excellent fit and was able to differentiate between patients with and without suicidal ideation^28^. This condensed version of the scale was found to maintain the integrity of the original measure while offering a streamlined approach to assessing hopelessness. Aloba and Akinsulore^12^ developed an alternative condensed 4-item Yoruba version of the BHS, featuring items 8, 9, 13, and 15, which they tested on a sample of 327 Nigerian adult psychiatric outpatients. The authors found that this shorter version of the BHS demonstrated satisfactory reliability and validity, on par with the longer form of the instrument. Forintos and colleagues^26^ validated a 3-item version of the BHS among a sample of Hungarian depressed patients. The scale comprised items with the highest correlations with the BHS total score and one item from the Beck Depression Inventory^29^.

The dimensionality of short versions of the BHS have predominantly been assessed using classical test theory (CTT). This method has certain shortcomings, namely that CTT may fail to capture variations in item difficulty and discrimination. Consequently, it may not fully capture the complexity and nuances of the scale's underlying structure, which could limit understanding of how the reduced number of items affects the assessment of hopelessness. For this reason, researchers^25,30^ have advocated for the use of statistical methods that offer more nuanced and complementary results. Recently, Balsamo and colleagues^25^ used receiver operating characteristics (ROC) and item response theory (IRT) to refine the BHS. These authors generated a 9-item unidimensional version of the BHS comprising Items 2, 6, 11, 12, 14, 16, 17, 18, and 20. The IRT-based 9-item version of the BHS demonstrated good discriminant validity and satisfactory reliability estimates among a sample of psychiatrically hospitalized adult patients^25^.

The current study aims to extend the work of Balsamo and colleagues^25^ by investigating the dimensionality of the 9-item BHS scale using a combination of Mokken Scale Analysis (MSA^31^) and CTT among a non-clinical sample. Mokken Scale Analysis can provide insight regarding the scalability and hierarchical ordering of items to identify whether the items form a single unidimensional scale. This approach helps in understanding whether the scale items consistently measure the same underlying construct. Meanwhile, MSA, as a form of IRT, delves into the difficulty and discrimination of individual items, offering a nuanced view of how respondents engage with the test^32,33^. In contrast, CTT is centered on overall test reliability and average item difficulty, but it does not account for individual item nuances^32^. By integrating MSA and CTT, this study offers a comprehensive methodological approach. MSA provides structural insights, establishing whether the BHS items form a coherent, unidimensional scale. Concurrently, CTT contributes a traditional view of the test's reliability, ensuring the study covers a broad spectrum of psychometric evaluation^33^.

This dual approach ensures a comprehensive psychometric evaluation, covering both the micro (item-level) and macro (test-level) aspects of the scale. It is particularly relevant in a non-clinical sample, as it allows for an examination of the scale's applicability and effectiveness in measuring varying levels of hopelessness in a general population, not just in clinical settings. In conclusion, the rationale for employing both MSA and CTT in this study lies in their complementary strengths. Together, they provide a robust framework for understanding the dimensionality and reliability of the BHS, ensuring that the scale is both theoretically sound and practically reliable for assessing hopelessness in a non-clinical context.

## Materials and methods

### Participants and procedure

We developed an online version of the questionnaires described in the Instruments section using Google Forms. As a rule of thumb, the literature suggests that an appropriate sample size for factor analysis should be based on the number of cases or the subjects-to-variable ratio (STV). In this regard Comrey and Lee^34^ suggests the following guideline: 100 = poor, 200 = fair, 300 = good, 500 = very good. In terms of STV ratio, Hair and colleagues^35^ suggests a ratio of 20 cases per item. Thus, at a minimum we required 180 respondents (20 cases X 9 items) but aimed for at least 300 to meet the guideline suggested by Comrey and Lee. Participants were students at a South African university in the Western Cape Province. To be included in the study participants had to be students registered at the institution. We used a random number generator to select a sample of 1,700 students and emailed the link to the online questionnaire to the selected students with a description of the study and an invitation to participate. Data collection took place in the period March–July 2022. A total of 322 university students responded positively, which represents a response rate of 18.9%.

The majority of the sample were women (77%), and most of the participants lived in an urban area (87.3%). The mean age of the sample was 26.01 years (SD = 10.19, range: 17–63). The study was conducted during the COVID-19 pandemic, and participants were asked to respond to two COVID-19-related questions. In response to these questions, 40.7% of participants indicated that they had lost a family member due to COVID-19 and 25.5% of participants had tested positive for COVID-19.

### Instruments

In addition to a brief demographic questionnaire, participants completed the following questionnaires: the BHS-9, the Satisfaction with Life Scale (SWLS)^36^, as well as short forms of the Center for Epidemiological Studies Depression Scale (CES-D10)^37^, and the trait scale of the Spielberger State-Trait Anxiety Inventory (STAI-T5)^38^.

The BHS-9 consists of nine items that measure hopelessness and pessimism about the future. Participants respond to each item using a dichotomous true/false scale. An example item of the BHS-9 is: “There’s no use in really trying to get anything I want because I probably won’t get it.” Balsamo and colleagues reported a Mokken scale reliability and alpha coefficient of 0.86 and found that the BHS-9 had good discriminant validity in being able being able to differentiate between psychiatric inpatients with a medium or high risk of suicide^25^.

The SWLS is a 5-item measure of the cognitive component of subjective well-being that represents participants’ cognitive evaluation of their life satisfaction. Responses to the five items are made on a 7-point scale with anchors of *Strongly disagree* (1) and *Strongly agree* (7). An example item of the SWLS is: “If I could live my life over, I would change almost nothing.” Diener and colleagues reported an estimate of internal consistency of 0.87, and the association between the SWLS and other measures of well-being serves as evidence of the tool’s validity. A South African study used a combination of CTT and IRT to examine the psychometric properties of the SWLS and confirmed the reliability, validity, and unidimensionality of the SWLS in the South African context^39^.

The CES-D10 consists of 10 items that measure depressive symptoms on a four-point scale ranging from *Rarely or none of the time* (0) to *Most or all of the time* (3). An example item of the CES-D10 is: “I had trouble keeping my mind on what I was doing.” Zhang and colleagues reported an alpha coefficient of 0.88 and demonstrated that the short version of the CES-D was as accurate as the original 20-item version in classifying participants with depressive symptoms^37^.

The STAI-T5 is a five-item measure of trait anxiety that is scored on a four-point scale with anchors of *Not at all* (1) and *Very much so* (4). An example item of the STAI-T5 is: “I worry too much over something that really doesn’t matter.” Zsido and colleagues reported a reliability coefficient of 0.86 for the STAI-T5, and the relationship between the STAI-T5 and measures of depression and life satisfaction serves as evidence of the tool’s external validity.

### Ethical considerations

The study was approved by the Humanities and Social Sciences Ethics Committee of the University of the Western Cape (Ethics Reference Number: HS22/2/9, February 2022) and conducted according to the guidelines of the Declaration of Helsinki. Participants provided informed consent, and participation was anonymous and voluntary.

### Data analysis

All items in the questionnaire were starred (i.e., all participants must complete them); thus, there were no missing values in the dataset. We used the “Mokken” package^40^ in R^41^ to conduct an MSA. We selected the double monotonicity model (DMM) of the MSA, which has four assumptions: unidimensionality, local independence, monotonicity, and invariant item ordering (IIO).

- Unidimensionality means that all the items of a scale are reflective of a single underlying latent variable. MSA uses an algorithm called automated item selection procedure (AISP) to indicate whether any item is unscalable (i.e., whether any item loads on the latent trait) and whether items are reflective of a single scale or multiple scales.
- Local independence means that an individual’s response to an item is not influenced by his or her response to any other item of the scale. Sijtsma and colleagues argue that unidimensionality implies local independence^42^; therefore, this assumption was not statistically tested in the current study.
- Monotonicity refers to the assumption that the probability of endorsing an item increases as the level of the latent trait increases. MSA provides an index called a *Crit* value to indicate whether an item violates the assumption of monotonicity. Researchers recommend that *Crit* values greater than 80 are considered a serious violation and *Crit* values less than 80 are considered a minor violation^43^.
- IIO refers to the assumption that the relative ordering of items in terms of endorsement or preference is the same across different respondents. For example, all respondents would find the item “I might as well give up because there is nothing I can do about making things better for myself” more intense than the item “I don’t expect to get what I really want.” In other words, IIO means that the order in which items are endorsed or responded to by individuals remains consistent across different levels of the latent trait being measured. MSA also provides a *Crit* value to evaluate violations of IIO. As with the assumption of monotonicity, a *Crit* value greater than 80 is indicative of a serious violation^43^. Where serious violations of IIO are identified, MSA provides an indication of which item should be excluded through the backward selection method.

MSA also provides a scalability coefficient for the total scale (*H*) and each individual item (*H*_*i*_). For the total scale, *H* is a measure of the strength of a scale such that an *H* coefficient from 0.30 to 0.39 reflects a weak scale, an *H* coefficient from 0.40 to 0.49 indicates medium strength, and an *H* coefficient greater than 0.5 indicates a strong scale^44^. For individual items, *H*_*i*_ indicates the extent to which the item contributes to the measurement of the latent trait (similar to item-total correlations). Items with *H*_*i*_ coefficients lower than 0.30 are considered weak items that do not contribute to the measurement of the latent trait^32^. MSA also provides an estimate of internal consistency referred to as Mokken scale reliability (*MS*_*rho*_) which ideally should be above 0.70^45^.

All classical test theory analyses were conducted using IBM SPSS for Windows version 28 (IBM Corp., Armonk, NY, USA). First, we used indices of skewness and kurtosis to examine the distribution of data. Skewness values between − 2 and + 2^35^ and excess kurtosis values that are between − 1 and + 1^46^ would indicate that the data are approximately normally distributed. By default, SPSS provide excess kurtosis which normalizes the kurtosis value by subtracting 3 from it^47^. In this regard references to kurtosis in the rest of the paper thus refers to excess kurtosis. We also obtained descriptive statistics (means and SDs) and reliabilities (alpha and omega) for all the scales. To further analyze the internal consistency and contribution of items to the measurement of the latent variable, we examined the inter-item correlations, item-total correlations, and factor loadings. Factor loadings were obtained by using exploratory factor analysis (EFA: principal components with varimax rotation). Prior to EFA, we examined the suitability of the data for factor analysis using the Kaiser–Meyer–Olkin (KMO) measure of sampling adequacy and Bartlett’s test of sphericity. A KMO greater than 0.50 and a significant Bartlett’s test result (*p* < 0.05) indicate that the sampling was adequate and that there is sufficient correlation between items to conduct factor analysis.

Inter-item correlations should ideally range from 0.15 to 0.85^48^; a finding lower than 0.15 indicates that the item does not reflect the same content domain as the other items and a finding greater than 0.85 indicates redundancy of items. In addition, average inter-item correlations should range from 0.15 to 0.50^49^. Item-total correlations from 0.30 to 0.70^50^ and factor loadings greater than 0.55^51^ reflect items that demonstrate a strong relationship with the underlying latent trait.

Finally, we examined the criterion-related validity of the short form of the BHS through the association (Pearson *r*) between hopelessness, as measured by the BHS, and various indices of psychological well-being (life satisfaction, depression, and anxiety).

## Results

The results of the MSA are reported in Table 1 and include AISP, *H*_*i*_ for the individual items of the BHS-9, standard error of *H*_*i*_, and *Crit* values for monotonicity and IIO.

**Table 1:** Mokken indices of the nine items of the Beck Hopelessness Scale (*n* = 322).

Item	N of factors resulting from AISP	*H*_*i*_	SE of *H*_*i*_	*Crit* value	
				monotonicity	IIO
2. Might as well give up	1	0.54	0.06	0	47
6. Expect to succeed in the future	1	0.41	0.09	25	89
11. Only unpleasantness ahead	1	0.56	0.05	0	6
12. Don’t expect to get what want	1	0.59	0.05	0	0
14. Things don’t work out	1	0.56	0.04	0	5
16. Never get what wants	1	0.61	0.04	0	40
17. Unlikely to get future satisfaction	1	0.60	0.04	0	35
18. Future seems vague	1	0.59	0.04	0	4
20. No use trying to get anything	1	0.62	0.05	0	46

AISP, Automated item selection procedure; *H*_*i*_, *H* coefficient of individual items; SE of *H*_*i*_, standard error of *H*_*i*_ coefficients; IIO, Invariant item ordering. Item numbers refer to the original numbering in the 20-item Beck Hopelessness Scale.

Table 1 indicates that AISP returned a value of 1 for all items, which indicates that the nine items represent a unidimensional scale. The *H* coefficient for the total scale was 0.57, which reflects a very strong unidimensional scale. *H*_*i*_ for all items was higher than 0.30 and ranged from 0.41 to 0.62. There was one minor violation of monotonicity for Item 6 (*Crit* value < 80). There were eight violations of IIO, but only the one related to Item 6 (“In the future, I expect to succeed in what concerns me most”) represented a significant and unacceptable violation (*Crit* value > 80). The backward selection method in MSA indicated that Item 6 should be removed from the scale. The results of the MSA for the remaining 8 items after the removal of Item 6 are reported in Online Appendix 1.

After the deletion of Item 6, the *H* coefficient for the total scale was 0.60, indicating a strong scale, and *H*_*i*_ coefficients ranged from 0.57 to 0.65. Compared to Table 1, the H-coefficients in Online Appendix Beck Hopelessness Scale showed marginal improvements. There were no violations of monotonicity for any of the eight items of the, and the *Crit* values for violations of IIO were all lower than 80 (highest *Crit* value = 15).

We subsequently examined the remaining eight items of the BHS using classical test theory, including EFA, inter-item correlations, and item-total correlations. KMO (0.90) and Bartlett’s test of sphericity (*p* < 0.001) confirmed that the data were suitable for factor analysis. EFA (with varimax rotation if more than one factor extracted) extracted one factor that accounted for 45.13% of the variance in the 9-item version and 48.86% of the variance in the 8-item version. Since the variance extracted was below 0.50, we used EFA to examine whether removal of any item would lead to an increase in the variance extracted. We found that the removal of item 18, “the future seems vague and uncertain to me” would increase the variance extracted to 51.77%. The inter-item correlations, item-total correlations, and factor loadings for the remaining seven items of the BHS as well as the item-total correlations and factor loadings for the 9-item solution are reported in Table 2.

**Table 2:** Classical test theory indices for the Beck Hopelessness Scale-7 (*n* = 322).

Item	2	11	12	14	16	17	20
2. Might as well give up	–						
11. Only unpleasantness ahead	0.39*	–					
12. Don’t expect to get what want	0.29*	0.40*	–				
14. Things don’t work out	0.33*	0.32*	0.37*	–			
16. Never get what wants	0.45*	0.49*	0.45*	0.39*	–		
17. Unlikely to get future satisfaction	0.42*	0.52*	0.44*	0.34*	0.54*	–	
20. No use trying to get anything	0.47*	0.55*	0.38*	0.36*	0.61*	0.56*	–
Mean	0.13	0.19	0.33	0.49	0.17	0.21	0.16
SD	0.34	0.39	0.47	0.50	0.38	0.41	0.37
Item-total correlation: 7 items	0.53*	0.61*	0.53*	0.47*	0.68*	0.65*	0.68*
Item-total correlation: 9 items	0.53*	0.61*	0.58*	0.47*	0.66*	0.65*	0.67*
Factor loadings: 7 items	0.66*	0.74*	0.65*	0.59*	0.79*	0.77*	0.80*
Factor loadings: 9 items	0.64*	0.73*	0.67*	0.58*	0.77*	0.76*	0.79*

Factor loadings for items excluded from 7-item scale: Item 6 = 0.44, Item 18 = 0.59. Item-total correlations for the two items: Item 6 = 0.34, Item 18 = 0.49.

*p < 0.001.

The inter-item correlations for the 7-item version were all significant (*p* < 0.001) and within an acceptable range (0.29–0.61). The average inter-item correlation was 0.41, which indicates that the items relate to the same content domain and there is no redundancy of items in the scale. The item-total correlations for the 7-item version were all significant and greater than 0.30 (range: 0.47–0.67), which indicates that all items contribute to the measurement of the latent variable. Similarly, the factor loadings were all significant (*p* < 0.001) and greater than 0.55 (range: 0.59–0.80), which demonstrates a strong association between the items and the underlying latent variable. However, in the case of the 9-item version the item-total correlation of Item 6, while meeting the acceptable criteria, was very low (0.33). Also, the factor loading for Item 6 in the EFA was less than 0.55, thus indicating that this item is not as strongly associated with the latent factor.

The results of a Mokken analysis of the 7-item version of the BHS are reported in Table 3.

**Table 3:** Mokken indices of the 7-item version of the Beck Hopelessness Scale.

Item	N of factors resulting from AISP	*H*_*i*_	SE of *H*_*i*_	*Crit* value	
				monotonicity	IIO
2. Might as well give up	1	0.55	0.06	0	0
11. Only unpleasantness ahead	1	0.56	0.05	0	0
12. Don’t expect to get what want	1	0.58	0.05	0	0
14. Things don’t work out	1	0.67	0.05	0	0
16. Never get what wants	1	0.62	0.04	0	4
17. Unlikely to get future satisfaction	1	0.60	0.05	0	4
20. No use trying to get anything	1	0.63	0.05	0	20

AISP, Automated item selection procedure; *H*_*i*_, *H* coefficient of individual items; SE of *H*_*i*_, Standard error of *H*_*i*_ coefficients; IIO, Invariant item ordering. Item numbers refer to the original numbering in the 20-item Beck Hopelessness Scale.

Table 3 reflects that all seven items loaded on a single scale which had an H-coefficient of 0.60 and there were no significant violations of monotonicity or IIO. The relationship between hopelessness as measured by the BHS-7 and other indices of psychological well-being is reported in Table 4, along with the reliabilities of all the scales and the descriptive statistics.

**Table 4:** Intercorrelations between variables, indices of skewness and kurtosis, and reliabilities of scales (*n* = 322).

Scale	1	2	3	4
1. Hopelessness	–			
2. Life satisfaction	− 0.50**	–		
3. Depression	0.49**	− 0.53**	–	
4. Anxiety	0.44**	− 0.41**	0.66**	–
Mean	1.69	19.35	14.15	12.36
SD	2.04	7.06	6.77	4.13
Skewness	1.28	− 0.03	0.05	0.03
Kurtosis	0.63	− 0.74	− 0.73	− 0.88
Alpha	0.83	0.86	0.84	0.88
Omega	0.83	0.86	0.85	0.88
Mokken scale reliability	0.85	–	–	–

**p < 0.001.

The indices of skewness were within the recommended range of − 2 to + 2 (− 0.03 to 1.28), and the indices of kurtosis were within the recommended range of − 1 to + 1 (− 0.88 to 0.63). These findings indicate that the data for all variables were approximately normally distributed. The internal consistency of all the scales may be considered satisfactory (α and ω: 0.83–0.88; MS_rho_ for hopelessness = 0.85).

The findings reported in Table 4 indicate that hopelessness was significantly negatively associated with life satisfaction (*r* =  − 0.53, *p* < 0.001, large effect) and significantly positively associated with depression (*r* = 0.51, *p* < 0.001, large effect) and anxiety (*r* = 0.47, *p* < 0.001, medium effect). Thus, high levels of depression are associated with low levels of life satisfaction and high levels of depression and anxiety.

## Discussion

The established association between hopelessness and adverse mental health outcomes, notably suicidality^8,9^, propelled the development of instruments capable of measuring this psychological construct. The BHS is the most widely used scale to assess hopelessness; however, the length of the original scale limited its utility, which has led to the development of shortened versions of the instrument^12,26^. The current study aimed to investigate the dimensionality of the 9-item version of the BHS^25^ among a non-clinical sample.

The AISP analysis demonstrated that the nine items of the BHS-9 form a unidimensional scale, confirming the findings of Balsamo and colleagues^25^. The high *H*_*i*_ coefficients (ranging from 0.57 to 0.63) indicate that the individual items contribute substantially to the overall measurement of hopelessness and confirm that these nine items collectively measure a single underlying construct. These coefficients reflect the degree to which the items are interrelated and collectively represent the latent variable.

A salient finding of our study was that Item 6 (“Expect to succeed in the future”) of the BHS-9 violated IIO. An IIO violation indicates that an item does not consistently match the difficulty or endorsement order compared to other items on the scale across varying levels. After removing Item 6 due to the IIO violation, the *H* coefficient for the total scale increased to 0.60. Item 6 also demonstrated a minor violation of monotonicity, which implies that as the underlying trait (i.e., severity of hopelessness) intensifies, endorsement of Item 6 does not consistently increase or stay constant. Respondents with a high degree of hopelessness would be expected to be more likely or as likely to endorse items that reflect that construct as those with a lesser degree of hopelessness. The *Crit* value indicated that the monotonicity violation related to Item 6 was minor. However, the violation of IIO indicates a complex relationship between Item 6 and the latent construct and suggests a need for further examination of the wording or conceptual relevance of this item. Notably, Item 6 is the sole item on the scale that is phrased positively. However, an EFA of the remaining eight items found that these eight items accounted for only 48.86% of the variance. There have been other studies that have reported variance explained values below 0.50 for other versions of the BHS. For example, Aloba and colleagues^52^ extracted two factors that accounted for 42.7% of the variance, while Bouvard and colleagues extracted one factor^53^ that accounted for 38.15% of the variance. However, extracted variance below 0.50 is considered to be indicative of a lack of convergent validity^54^ and we thus ran a number of EFA’s to determine whether removal of any of the items would lead to an increase in variance explained. EFA demonstrated that the removal of item 18, “the future seems vague and uncertain to me” would increase the variance explained to an acceptable 51.77%.

Our results suggest that the remaining seven items of the BHS are more strongly interrelated than the original nine items, enhancing the scale's reliability. The EFA confirmed the unidimensionality of the BHS-7, with a single factor accounting for a significant portion (51.77%) of the variance in the items. This finding further corroborates the scale's ability to capture hopelessness as a single construct. Inter-item correlations, item-total correlations, and factor loadings all support the internal consistency and coherence of the scale, which indicates that the items measure the same underlying concept and contribute consistently to the measurement. The reported associations between hopelessness and other psychological indices provide valuable evidence of the scale's criterion-related validity. The relationships observed between scores on the hopelessness scale and other well-established psychological measures indicate that the scale effectively measures what it purports to measure. The significant negative correlation with life satisfaction and positive correlations with depression and anxiety align with theoretical expectations. These findings suggest that the BHS-7 effectively captures the essence of hopelessness as it relates to these key psychological factors.

The revision and generation of a 7-item version of the BHS (following the removal of item 6 due to its violations of IIO and monotonicity as well as item 18 to increase variance extracted) result in a more refined tool for the assessment of hopelessness. This improvement is evident in several key statistical measures. First and foremost, the removal of Item 6 resulted in a marginal increase in the overall H-coefficient for the revised 7-item version of the scale, indicating a more cohesive measure of hopelessness. Similarly, the individual H-coefficients for each item in the 7-item scale also showed an increase compared to the 9-item version, suggesting that each item more effectively contributes to the overall construct of hopelessness.

Furthermore, the 7-item version demonstrated superior compliance with the assumptions of monotonicity and IIO. Unlike the 9-item version, the revised scale exhibited no violations of monotonicity and no serious violations of IIO, signifying a more reliable and valid measure. In terms of EFA, the 7-item version accounted for a higher percentage of variance in the items (51.77%) compared to the 9-item version (45.13%). This increase in variance explained by the 7-item version suggests a more coherent and unified construct of hopelessness.

Additionally, the statistical performance of Item 6 itself further justified its removal. The item-total correlation for Item 6 was 0.33, hovering very close to the lower threshold of acceptability (0.30), indicating a weaker relationship with the overall scale compared to other items. Moreover, the factor loading of Item 6, at 0.44, was below the generally accepted level of 0.55, highlighting its relatively lower contribution to the scale's construct.

In summary, the exclusion of items 6 and 18 from the BHS rectified specific statistical shortcomings and resulted in a more coherent and statistically sound scale. The 7-item version emerged as a more robust tool for measuring hopelessness, with improved internal consistency and factor structure, reinforcing its utility in both research and clinical settings.

Reducing a scale's length while preserving (or enhancing) its psychometric properties can be advantageous for both research and clinical settings. A shorter yet equally effective scale offers faster administration, reduced participant fatigue, and potentially higher response rates, especially in contexts where time or respondent attention is limited. Its shortened form makes it more feasible for use in diverse settings, including primary care, community mental health programs, and even in non-clinical environments such as workplaces or educational institutions. This increased usability can lead to more widespread and routine screening for hopelessness, facilitating early identification of individuals at risk of adverse mental health outcomes, including depression and suicidality.

In clinical practice, the BHS-7 can be integrated into patient assessments to quickly and effectively gauge levels of hopelessness, allowing healthcare providers to tailor interventions more precisely. For instance, in therapy settings, clinicians can use the scale to monitor changes in hopelessness over time, helping to evaluate the effectiveness of treatment approaches. In research, the scale's enhanced psychometric properties and shorter length can improve participant engagement and response rates, leading to more robust data collection and analysis.

Furthermore, the potential applicability of the BHS-7 to a broader, non-clinical population makes it a valuable tool for preventative mental health initiatives. Community mental health programs aimed at generating awareness, reducing mental health stigma and preventing suicide can employ the scale to identify at-risk individuals who might not typically seek psychiatric help. Educational campaigns and interventions can be designed around the insights gained from the scale, promoting mental well-being and resilience against hopelessness.

The BHS-9 was formulated based on a clinical population of psychiatrically hospitalized adult patients predominantly diagnosed with Bipolar II Disorder. The severity of Bipolar II Disorder, especially within the context of psychiatric hospitalization, inherently implies nuanced expressions of hopelessness that may be particularly intense in this population group. Conversely, the BHS-7 was derived from a non-clinical sample that encapsulates a broad spectrum of the general population. Thus, the findings may capture subtleties and variations of hopelessness that may not be as pronounced or may manifest differently in clinical settings. The BHS-7 serves as an important step in advancing the assessment tools used for hopelessness and ensuring they are both practical and theoretically sound.

While the provided results offer novel insights, there are certain limitations to the current study. Although the BHS-7 was derived from a non-clinical sample, it is essential to acknowledge that findings from one segment of the general population might not seamlessly translate to all subgroups or cultural contexts. The study may not have captured nuances of hopelessness that vary based on socio-economic, ethnic, or other demographic factors.

An important limitation of our study relates to the lack of sociodemographic information for both the contacted and the responder samples. This limitation stems from strict adherence to the Protection of Personal Information Act, which significantly constrained our ability to obtain personal information, including email addresses and detailed demographic data. Due to these legal restrictions, the invitation to participate in our study was centrally distributed by the Registrar’s office without the researchers having any insight into the specific demographics of the individuals in the randomly selected sample. This approach, while necessary to comply with privacy regulations, inevitably limits our understanding of the selection bias that may have occurred and hinders our ability to fully ascertain the representativeness of our sample and, by extension, the generalizability of our findings.

Additionally, the study was undertaken in the context of the COVID-19 pandemic, a factor that could significantly influence participants' responses. This unique context may have influenced the way participants perceived and responded to the BHS items, potentially affecting the generalizability of the findings to non-pandemic circumstances.

While the removal of Item 6 due to violations of IIO is justified, it may also lead to potential loss of certain aspects or expressions of hopelessness that the original item might have captured. Like many psychological scales, the BHS relies on self-reported data, which can be influenced by social desirability bias, recall bias, or varying levels of introspective accuracy among participants. The cross-sectional nature of the data limits the ability to track changes in feelings of hopelessness over time. Future research of a longitudinal nature among more diverse samples could be beneficial to address these limitations. Longitudinal studies could reveal the scale's sensitivity to changes in hopelessness levels over time and its predictive validity. It is also advisable to undertake comparative analysis across diverse populations to determine which version of the BHS (e.g., BHS-7 or BHS-8) is more appropriate in that setting. Conducting qualitative research to explore participants' understanding of individual scale items, particularly those with violations of monotonicity or IIO, could reveal insights into the conceptual meaning of those items. Finally, a salient limitation of the current study pertains to the response rate and potential attrition bias which may limit the representativeness of our findings. The high rate of non-response could introduce response bias, where the characteristics or attitudes of those who chose to participate differ significantly from those who did not. The attrition rate may have also affected the diversity of our sample. This skewed representation can limit the generalizability of our findings to the broader student population.

## Conclusion

The reported results provide a comprehensive understanding of the psychometric properties of the BHS-7, shedding light on its unidimensionality, reliability, validity, and associations with other psychological constructs. The shortened scale demonstrates strong potential; however, further research is needed to address the current study limitations and enhance the scale's applicability across diverse populations and contexts.

### Supplementary Information

Supplementary Information.

## Data Availability

The raw data supporting the conclusions of this article will be made available by the authors without undue reservation. Contact tpretorius@uwc.ac.za.
