# Supplementary material for: A unidimensional short form of the Beck Hopelessness Scale (BHS-7) derived using item response theory
Source: Sci Rep. 2024 Mar 12;14:6021. doi: 10.1038/s41598-024-56792-x (PMC10933447; doi:10.1038/s41598-024-56792-x)
Supplement: Supplementary file 1 — Supplementary Information. [file 41598_2024_56792_MOESM1_ESM.docx]

**Appendix 1: Mokken indices of the 8-item version of the Beck Hopelessness Scale**

| **Item** | **N of factors** | ***H_i_*** | **SE of *H_i_*** | ***Crit* value** | |
| --- | --- | --- | --- | --- | --- |
|  | **resulting** |  |  | **monotonicity** | **IIO** |
|  | from AISP |  |  |  |  |
| 2. Might as well give up | 1 | 0.57 | 0.06 | 0 | 6 |
| 11. Only unpleasantness ahead | 1 | 0.58 | 0.05 | 0 | 6 |
| 12. Don’t expect to get what want | 1 | 0.59 | 0.05 | 0 | 0 |
| 14. Things don’t work out | 1 | 0.58 | 0.04 | 0 | 6 |
| 16. Never get what wants | 1 | 0.63 | 0.04 | 0 | 6 |
| 17. Unlikely to get future satisfaction | 1 | 0.61 | 0.04 | 0 | 5 |
| 18. Future seems vague | 1 | 0.59 | 0.04 | 0 | 15 |
| 20. No use trying to get anything | 1 | 0.65 | 0.05 | 0 | 3 |

*Note.* AISP = automated item selection procedure, *H_i_* = *H* coefficient of individual items, SE of *H_i_* = standard error of *H_i_* coefficients, IIO = invariant item ordering. Item numbers refer to the original numbering in the 20-item Beck Hopelessness Scale.
